# Latent profile and influencing factors of volume management behaviors in patients with chronic heart failure: a cross-sectional study

**DOI:** 10.3389/fcvm.2025.1682875

**Published:** 2025-10-10

**Authors:** Lu Chen, Rui Wu, Huiwen Wang, Yinjie Li, Mengdie Liu, Hua Chen, Dan Xiao, Xiaoyun Xiong

**Affiliations:** ^1^ The Second Affiliated Hospital, Jiangxi Medical College, Nanchang University, Nanchang, China; ^2^ School of Nursing, Jiangxi Medical College, Nanchang University, Nanchang, China

**Keywords:** chronic heart failure, volume management, latent profile, influencing factors, cross-sectional study

## Abstract

**Aims:**

This study aimed to identify latent profiles of volume management behaviors among patients with chronic heart failure using latent profile analysis and to explore the factors influencing different behavioral profiles.

**Methods:**

A total of 381 patients with chronic heart failure were recruited through convenience sampling from the department of cardiovascular medicine at a tertiary hospital in Nanchang City, between December 2024 and May 2025. Data were collected using the General Information Questionnaire, the Home Volume Management Self-Rating Scale for Patients with Chronic Heart Failure, the Self-Efficacy for Managing Chronic Disease 6-Item Scale, and the Social Support Rating Scale. Latent profile analysis was conducted on the volume management behaviors of patients with chronic heart failure, and multinomial logistic regression analysis was used to examine the factors influencing the different latent profiles.

**Results:**

Three distinct latent profiles of volume management behavior were identified: “low capacity-vulnerable type” (39.9%), “high capacity-robust type” (15.5%), and “moderate capacity-dependent type” (44.6%). Multivariate logistic regression analysis revealed that educational level, duration of disease, social support, and self-efficacy were factors influencing the latent profiles of volume management behaviors in chronic heart failure.

**Conclusion:**

Overall, volume management behaviors in patients with chronic heart failure were suboptimal, with notable variation across different profiles. Tailored interventions based on these profile characteristics and influencing factors may enhance volume management abilities in this population.

## 1 Introduction

Chronic heart failure (CHF) is a complex clinical syndrome characterized by persistent cardiac dysfunction and progressive deterioration (1). It is associated with high mortality and readmission rates and is currently the only cardiac condition exhibiting an upward trend (2, 3). According to reports, the global prevalence of heart failure is estimated to range from 1% to 3%, affecting over 64 million individuals worldwide (4). In China, the burden of CHF is particularly severe, with 8.9 million affected individuals and a standardized prevalence rate of 1.1% (5, 6). Furthermore, approximately 3 million new cases are diagnosed each year (7).

Volume overload is a key pathological mechanism in the onset and progression of CHF, clinically presenting as pulmonary or systemic congestion and inadequate tissue perfusion (8). It is a major contributor to recurrent hospitalizations and poor prognosis after patient discharge (9). Evidence suggests that volume overload often occurs before the onset of congestive symptoms and signs (10). Without timely intervention, it may result in compensatory fluid redistribution, interstitial fluid retention, and multi-organ dysfunction, ultimately advancing to the decompensated stage of heart failure (11). Therefore, early initiation of volume management is essential for patients with CHF.

Although there is no universally accepted definition of volume management in CHF patients, it generally refers to the dynamic assessment of volume status and the implementation of appropriate measures to maintain optimal fluid balance (12). In patients with CHF, volume management encompasses several key aspects, including monitoring of volume status, fluid control, diuretic management, and sodium restriction (13). Current guidelines recommend volume management as a key strategy in CHF disease management, with the primary goals of reducing cardiac workload cardiac workload, stabilizing hemodynamic status, and achieving optimal fluid balance (14).

In China, many CHF patients exhibit inadequate volume management behaviors following discharge, posing substantial challenges for clinical implementation (15). Personalized interventions provide an effective strategy to enhance these behaviors. However, previous studies have primarily employed variable-centered methods, treating participants as a homogeneous group (16). This approach overlooks the latent heterogeneity within populations, thereby failing to account for variations in behavioral patterns across distinct patient groups. As a result, the specificity and effectiveness of interventions are compromised. Latent Profile Analysis (LPA) is an “individual-centered” statistical method that groups participants with similar response patterns on questionnaire items into the same latent category (17). Unlike traditional cluster analysis or factor analysis, LPA more effectively identifies heterogeneity within groups and uncovers complex behavioral patterns, providing more precise classification results for health behavior research (18).

Social Cognitive Theory, proposed by American psychologist Albert Bandura in the 1980s, emphasizes that individual behavior is shaped by the interaction of personal, behavioral, and environmental factors (19). Previous studies have shown that self-efficacy, a core personal factor, can influence patients’ behavioral confidence, while social support, a key environmental factor, can have a positive motivational effect on their behavior (20). Therefore, this study examines the impact of two key variables, self-efficacy and social support, on volume management behaviors in patients with CHF, grounded in social cognitive theory. Additionally, LPA is used to identify latent categories of volume management behaviors and explore variations in influencing factors across distinct subgroups. The results aim to provide both theoretical and practical foundations for the development of more targeted and personalized volume management interventions.

## 2 Materials and method

### 2.1 Participants

This study adopted a convenience sampling approach to recruit patients with chronic heart failure from a tertiary hospital in Nanchang, China, between December 2024 and May 2025.

Inclusion criteria were as follows:

1. Patients who met the diagnostic criteria of the “Chinese Guidelines for the Diagnosis and Treatment of Heart Failure 2024” and were diagnosed with chronic heart failure (14);
2. Age ≥18 years;
3. Clear consciousness with no cognitive or communication impairments;
4. Willingness to participate in the study.

Exclusion criteria included:

1. No prior use of diuretics;
2. Patients with chronic heart failure who have hemodynamic instability or are in the acute decompensation stage;
3. Presence of severe dysfunction in vital organs (e.g., severe cardiac, hepatic, or renal insufficiency);
4. Diagnosis of mental illness or intellectual disability.

### 2.2 Sample size

This study employs Latent Profile Analysis (LPA) as the statistical method. Previous literature indicates that when the sample size for LPA is fewer than 300, the risk of poor model fit and convergence issues increases (21). Therefore, a minimum sample size of at least 300 was established. Considering a 20% attrition rate, the final required sample size was determined to be 375 participants.

### 2.3 Measures

#### 2.3.1 General information questionnaire

Developed by the research team based on a literature review, this questionnaire consists of two sections: (1) Sociodemographic characteristics: gender, age, marital status, educational level, cohabitation situation, place of residence, employment status, monthly income, and payment pattern; (2) Disease-related information: NYHA classification, comorbidity, left ventricular ejection fraction (LVEF), duration of disease, body mass index (BMI), brain natriuretic peptide (BNP), smoking, and drinking alcohol.

#### 2.3.2 Home volume management self-rating scale for patients with chronic heart failure

Developed by Ye Linbin in China, this 27-item scale assesses volume management behaviors in patients with chronic heart failure across four dimensions: self-evaluation, self-maintenance, self-management, and self-confidence (22). Each item is rated on a 5-point Likert scale, with total scores ranging from 27 to 135. Higher scores indicate a better ability to manage volume. In this study, the scale demonstrated good internal consistency, with a Cronbach's alpha of 0.896.

#### 2.3.3 Social support rating scale (SSRS)

This scale, developed by Xiao Shuiyuan in 1986, is designed to assess individuals’ social support status (23). The questionnaire comprises 10 items: items 1–4 and 8–10 are single-choice questions, each scored from 1 to 4 points; item 5 consists of 5 sub-items, with each scored from 1 to 4 points based on the level of support; items 6 and 7 are scored according to the number of support sources. The scale encompasses three dimensions: subjective support, objective support, and utilization of social support. The total score is the sum of scores across all dimensions, with higher scores indicating greater levels of social support. Typically, a total score below 22 indicates low social support, 22–45 indicates a moderate level of social support, and a score of 45 or above indicates a relatively satisfactory level of social support. In this study, the Cronbach's alpha coefficient for this scale was 0.858.

#### 2.3.4 Self-Efficacy for managing chronic disease 6-item scale (SEMCD-6)

This scale, developed by Lorig at Stanford University, is designed to assess self-efficacy among patients with chronic diseases (24). The Chinese version was translated by Zhang Meixia in 2022 and underwent cross-cultural adaptation and validation for the Chinese population (25). It consists of six items across two dimensions: symptom management and general disease management. Each item is rated from 1 (not at all confident) to 10 (completely confident). The mean score across the six items reflects the level of self-efficacy, with higher scores indicating greater self-efficacy. In this study, the scale demonstrated a Cronbach's alpha coefficient of 0.891.

### 2.4 Data collection

All researchers involved in the study received standardized training prior to commencing the survey. The same cardiologist, who has more than five years of clinical experience, was responsible for assessing the health status of all target patients. After the assessment, three trained researchers, in collaboration with ward nurses, collected sociodemographic information and evaluated the patients’ volume management capability, social support, and self-efficacy at the bedside. The researchers also explained the purpose and significance of the study at the outset to obtain informed consent from the participants. For participants with difficulty Reading and writing, the researchers read the questions, clarified the content of each item, and completed the questionnaire based on the patients’ responses. After the survey, the researchers reviewed the questionnaires on-site to ensure that no omissions or errors were present

### 2.5 Statistical analysis

After data verification and double entry by two independent researchers, latent profile analysis was conducted using Mplus8.3. The average scores of the four dimensions from the Home Volume Management Self-Rating Scale for Patients with Chronic Heart Failure were used as indicator variables. The model selection process began with a baseline model containing a single class, and the number of latent classes was progressively increased. Model selection criteria based on literature (21, 26): (1) Akaike Information Criterion (AIC), Bayesian Information Criterion (BIC), and Adjusted BIC (aBIC): Lower values indicate better model fit. (2) Entropy: Ranging from 0 to 1, higher values indicate greater classification accuracy. An entropy ≥0.80 suggests good class separation. (3) Lo-Mendell-Rubin Likelihood Ratio Test (LMR) and Bootstrap Likelihood Ratio Test (BLRT): A *p*-value < 0.05 indicates that the model with k classes fits significantly better than the model with k-1 classes.

After determining the optimal classification model, this study employed SPSS 27.0 for statistical analysis. Before conducting descriptive statistics, continuous variables underwent normality testing. Normally distributed continuous variables were expressed as mean ± standard deviation, with intergroup comparisons conducted using one-way analysis of variance (ANOVA). Non-normally distributed continuous variables were represented by the median and interquartile range, with intergroup comparisons performed using the Kruskal–Wallis H test. Categorical variables were presented as frequencies and percentages, with intergroup comparisons performed using chi-square tests or Fisher's exact test. Finally, variables that demonstrated statistical significance in univariate analysis were included in multivariate logistic regression analysis to explore the influencing factors across different categories further.

## 3 Results

### 3.1 Common method bias test

Because the measurement method in this study consisted only of questionnaire surveys, we used the Harman single-factor test to assess common method bias. Ten factors had eigenvalues greater than 1, and the variance explained by the largest single factor was 24.129%, which is below the critical threshold of 40% (27). These results indicate that common method bias is not a serious concern in this study.

### 3.2 Latent profile analysis of volume management behaviors in patients with chronic heart failure

Table 1 presents the model fitting statistics for four potential categories. The entropy value of Model 3 is the highest, at 0.872, and both LMR and BLRT reach significant levels, indicating strong goodness of fit and accuracy. Although LMR and BLRT are significant in Model 2, their entropy value is lower than that of Model 3, suggesting lower accuracy. The LMR value of Model 4 is not statistically significant. Therefore, Model 3 is selected as the optimal model.

**Table 1:** Model fit indices for the compared latent profiles (*n* = 381).

Model	AIC	BIC	aBIC	Entropy	BLRT	LMR	Class Probability
1	2,581.418	2,612.96	2,587.578	–	–	–	–
2	2,121.297	2,172.553	2,131.307	0.829	<0.001	0.030	0.738/0.262
3	1,890.607	1,961.577	1,904.466	0.872	<0.001	<0.001	0.402/0.155/0.444
4	1,837.057	1,927.741	1,854.766	0.869	<0.001	0.054	0.394/0.430/0.136/0.039

AIC, akaike information criteria; BIC, bayesian information criteria; aBIC, adjusted bayesian information criteria; BLRT, bootstrapped likelihood ratio test; LMR, Lo-Mendell-Rubin likelihood ratio test; *P* < 0.05.

**Figure 1:**
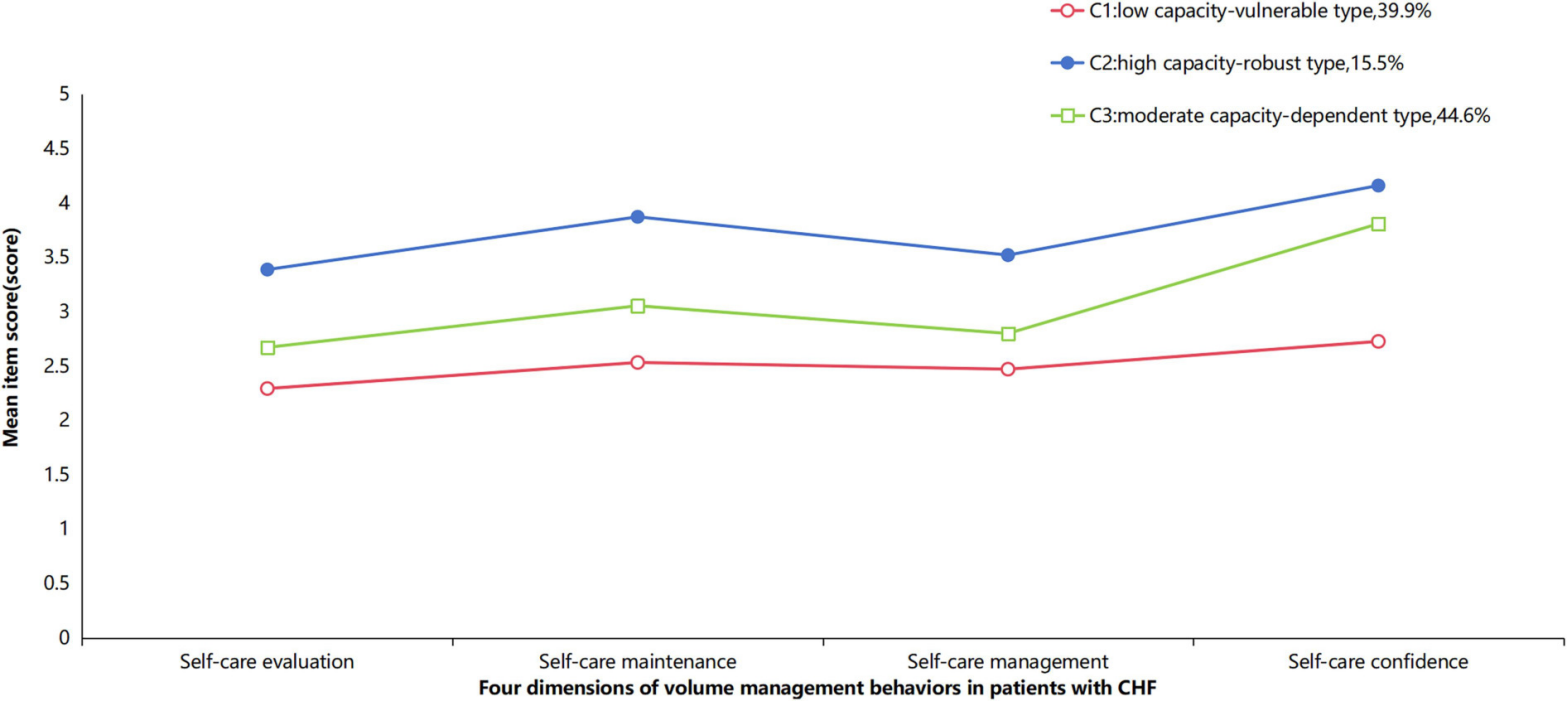
The characteristic distribution of three latent profiles of volume management behaviors in patients with chronic heart failure.

Based on the three latent profiles of volume management behaviors in patients with chronic heart failure, a category graph was constructed, and the categories were named according to their characteristics (see Figure 1). The first category, characterized by the lowest average score across all dimensions, is labeled “low capacity-vulnerable type”. The second category, with relatively high scores in all dimensions, is named “high capacity-robust type”. The third category, which displays medium-level scores in the first three dimensions and a higher score in self-care confidence, reflects patients who lack scientific guidance for volume management and depend more on medical professionals’ instructions to take appropriate actions. This category is named “moderate capacity-dependent type”.

### 3.3 Comparison of demographic variables across latent profiles

Initially, 400 patients were surveyed. Nineteen invalid questionnaires were excluded (10 patients underwent two assessments, and 9 patients had incomplete information). Ultimately, valid data from 381 patients were included in the statistical analysis, resulting in a questionnaire response rate of 95.25%. The data collection exceeded the originally planned sample size to ensure both data quality and completeness while increasing the sample size to better meet analytical requirements. Univariate analysis revealed significant differences across the three latent profiles in terms of educational level, gender, duration of disease, place of residence, Employment status, monthly income, self-efficacy, and social support (*P* < 0.05) (see Table 2).

**Table 2:** Overview of demographic characteristics of volume management behaviors in patients with chronic heart failure (*n* = 381).

Variables	Total number (*n* = 381)	Class 1 (*n* = 153)	Class 2 (*n* = 59)	Class 3 (*n* = 169)	*χ*^2^/F/H	*P*
Age (years)	70.69 ± 11.087	71.67 ± 9.73	72.59 ± 10.478	69.12 ± 12.241	3.016^b^	0.052
Gender					14.210^a^	<0.001
Male	223 (58.5%)	72 (47.1%)	41 (69.5%)	110 (65.1%)		
Female	158 (41.5%)	81 (52.9%)	18 (30.5%)	59 (34.9%)		
Marital status					8.317^a^	0.081
Married	295 (77.4%)	111 (72.5%)	50 (84.7%)	134 (79.3%)		
Divorced/widowed	81 (21.3%)	41 (26.8%)	7 (11.9%)	33 (19.5%)		
Unmarried	5 (1.3%)	1 (0.7%)	2 (3.4%)	2 (1.2%)		
Educational level					93.024^a^	<0.001
Elementary school and under	198 (52.0%)	123 (80.4%)	15 (25.4%)	60 (35.5%)		
Middle school	81 (21.3%)	20 (13.1%)	13 (22.0%)	48 (28.4%)		
High school and above	102 (26.8%)	10 (6.5%)	31 (52.5%)	61 (36.1%)		
Cohabitation situation					4.930^a^	0.086
Live with others	341 (89.5%)	132 (86.3%)	57 (96.6%)	152 (89.9%)		
Living alone	40 (10.5%)	21 (13.7%)	2 (3.4%)	17 (10.1%)		
Place of residence					52.272^a^	<0.001
City	255 (66.9%)	70 (45.8%)	50 (84.7%)	135 (79.9%)		
Rural area	126 (33.1%)	83 (54.2%)	9 (15.3%)	34 (20.1%)		
Payment pattern					4.777^a^	0.311
Medical insurance for rural residents	200 (52.5%)	89 (58.2%)	32 (54.2%)	79 (46.7%)		
Medical insurance for urban workers	161 (42.3%)	57 (37.3%)	23 (39%)	81 (47.9%)		
Self-expense	20 (5.2%)	7 (4.6%)	4 (6.8%)	9 (5.3%)		
Employment status					46.590^a^	<0.001
Employed	34 (8.9%)	7 (4.6%)	5 (8.5%)	22 (13%)		
Retirement	218 (57.2%)	65 (42.5%)	46 (78%)	107 (63.3%)		
Freelancer	129 (33.9%)	81 (52.9%)	8 (13.6%)	40 (23.7%)		
Monthly income(RMB)					75.743^a^	<0.001
<2,000	52 (13.6%)	31 (20.3%)	3 (5.1%)	18 (10.7%)		
2,000–5,000	168 (44.1%)	98 (64.1%)	17 (28.8)	53 (31.45%)		
>5,000	161 (42.3%)	24 (15.7%)	39 (66.1%)	98 (58.0%)		
Smoking					0.782^a^	0.941
Yes	55 (14.4%)	23 (15%)	7 (11.9%)	25 (14.8%)		
No	267 (70.1%)	107 (69.9%)	41 (69.5%)	119 (70.4%)		
Quit smoking	59 (15.5%)	23 (15%)	11 (18.6%)	25 (14.8%)		
Drinking alcohol					2.605^a^	0.626
Yes	36 (9.4%)	14 (9.2%)	3 (5.1%)	19 (11.2%)		
No	283 (74.3%)	113 (73.9%)	48 (81.4%)	122 (72.2%)		
Quit drinking	62 (16.3%)	26 (17%)	8 (13.6%)	28 (16.6%)		
Course of disease (years)					143.437^a^	<0.001
<1	131 (34.4%)	40 (26.1%)	4 (6.8%)	87 (51.5%)		
1–5	161 (42.3%)	85 (55.6%)	29 (49.2%)	47 (27.8%)		
>5	89 (23.4%)	28 (18.3%)	26 (44.1%)	35 (20.7%)		
Comorbidity					6.281^a^	0.179
1–2	27 (7.1%)	8 (5.2%)	5 (8.5%)	14 (8.3%)		
3–4	65 (17.1%)	33 (21.6%)	5 (8.5%)	27 (16.0%)		
≥5	289 (75.9%)	112 (73.2%)	49 (83.1%)	128 (75.7%)		
NYHA classification					8.416^a^	0.077
II-class	194 (50.9%)	74 (48.4%)	24 (40.7%)	96 (56.8%)		
III-class	152 (39.9%)	66 (43.1%)	25 (42.4%)	61 (36.1%)		
IV-class	35 (9.2%)	13 (8.5%)	10 (16.9%)	12 (7.1%)		
BNP	1,084.00	1,025.00	1,825.00	1,047.00	3.901^c^	0.142
	(392.00, 3,087.00)	(460.50, 2,482.50)	(469.00, 4,610.00)	(328.00, 3,085.50)		
LVEF	48.00 ± 13.73	48.92 ± 12.73	47.36 ± 13.321	47.40 ± 14.74	0.570^b^	0.566
BMI	23.16 ± 3.87	22.62 ± 3.69	23.42 ± 4.86	23.53 ± 3.62	2.252^b^	0.107
SSRS	29.58 ± 5.12	26.07 ± 3.82	33.58 ± 4.91	31.37 ± 4.197	97.022^b^	<0.001
SEMCD-6	5.51 ± 1.27	4.90 ± 1.08	6.34 ± 1.61	5.78 ± 1.02	37.725^b^	<0.001

^a^ *χ*^2^; ^b^F; ^c^H.

### 3.4 Multivariate analysis of characteristics in potential profiles of volume management behaviors in chronic heart failure patients

Using the latent profile of volume management behaviors in patients with chronic heart failure as the dependent variable, and the variables identified as significant in the univariate analysis as independent variables, with “low capacity—vulnerable type” as the reference group, multivariate analysis was conducted. The results indicated that educational level, self-efficacy, social support, and duration of disease are the primary factors influencing the potential categories of volume management behaviors in these patients, as shown in Table 3.

**Table 3:** Multinomial logistic regression analysis of the latent categories of volume management of patients with chronic heart failure.

Items	*β*	SE	Wald*χ*^2^	*P*	OR	95%Cl
C1 vs. C2						
SSRS	0.525	0.074	49.895	<0.001	1.691	(1.462, 1.957)
SEMCD-6	0.701	0.216	10.577	0.001	2.017	(1.321, 3.078)
Course of disease						
<1	−4.643	0.808	33.013	<0.001	0.010	(0.002, 0.047)
1–5	−2.691	0.573	22.035	<0.001	0.068	(0.022, 0.209)
Education level						
Elementary school and under	−2.674	0.734	13.255	<0.001	0.069	(0.016, 0.291)
Middle school	−2.036	0.727	7.837	0.005	0.119	(0.031, 0.543)
C1 vs. C3						
SSRS	0.351	0.058	36.224	<0.001	1.420	(1.267, 1.592)
SEMCD-6	0.417	0.154	7.342	0.007	1.517	(1.122, 2.051)
Course of disease						
<1	−1.034	0.491	4.430	0.035	0.356	(0.136, 0.931)
1–5	−1.961	0.463	17.951	<0.001	0.141	(0.057, 0.349)
Education level						
Elementary school and under	−2.088	0.570	13.426	<0.001	0.124	(0.041, 0.379)
Middle school	−1.233	0.580	4.517	0.034	0.291	(0.094, 0.909)

## 4 Discussion

### 4.1 Three latent profiles of volume management behaviors in patients with chronic heart failure

To our knowledge, this is the first study to explore the heterogeneity of volume management behaviors in patients with chronic heart failure. Additionally, we investigated the impact of factors such as sociodemographic characteristics, self-efficacy, and social support on these subgroups. The results of this study demonstrate significant heterogeneity in the volume management behaviors of patients with CHF. Based on model fitting results, we classified these behaviors into three categories: “low capacity-vulnerable type”, “high capacity-robust type”, and “moderate capacity-dependent type”.

Patients in Group C1 represented 39.9% of the total sample, scoring the lowest across all four dimensions of volume management, which indicates overall poor volume management capabilities in this group. This finding aligns with previous research suggesting that multiple factors hinder patients’ effective implementation of volume management (28). Our study further refines the understanding of subgroup characteristics related to volume management. Based on our findings, the underlying factors may be linked to lower educational levels and inadequate knowledge of volume management. Additionally, this group exhibited relatively poor social support and self-efficacy, further undermining their confidence in managing volume (29). Therefore, healthcare providers should place greater emphasis on this vulnerable population and offer enhanced support.

Group C2 comprised 15.5% of the sample. This group outperformed the other two groups across all dimensions of volume management, particularly excelling in self-care confidence, suggesting that these patients possess strong volume management abilities. Notably, this group had the smallest proportion of participants. One possible explanation is that the study population consisted primarily of elderly individuals, most of whom grew up under the unique socio-cultural and educational conditions of mid-20th-century China. As a result, the proportion of participants who had received higher education was relatively low (30). Additionally, this study found that patients in Group C2 benefit from greater psychosocial resources, such as social support and self-efficacy, which enhance their confidence in disease management and adherence to behavioral guidelines. Therefore, future efforts should focus on fully leveraging the positive traits of this patient group while exploring replicable experiences to provide role models and guidance for other patients with weaker conditions.

Group C3 comprised 44.6% of the participants, making it the largest group. This suggests that the volume management capacity of most patients with CHF is at a moderate level. Characteristic analysis reveals that this group scored relatively low in the first three dimensions, but exhibited high self-care confidence, indicating that these patients have a certain level of confidence in volume management. However, due to insufficient scientific guidance, this confidence has not been effectively translated into practical operational skills. This may be attributed to the relatively short disease duration of the patients, along with a lack of sufficient knowledge and experience in volume management (31). Therefore, intervention strategies for this group should focus on converting confidence into actionable practice. Early implementation of scientific, standardized education is crucial in helping patients master volume management skills, thereby facilitating their transition from passive dependence to active coping.

### 4.2 Influencing factors of the latent profile of volume management behaviors

#### 4.2.1 Educational level

The findings of this study demonstrate that educational attainment influences the latent profiles of volume management behaviors among patients with CHF. Compared with patients classified as “low capacity-vulnerable type”, those with higher levels of education were more likely to be categorized as “high capacity–robust type” or “moderate capacity-dependent type.” Higher educational attainment is often associated with greater health literacy, which enables patients to access, comprehend, and apply health information more effectively (32). This enhanced capacity improves disease awareness and decision-making, thereby facilitating accurate assessment of fluid status and the adoption of appropriate management strategies. In contrast, patients with lower educational attainment may face challenges in acquiring and processing health-related information, making it difficult for them to systematically master disease knowledge and volume management skills (33). Consequently, they may experience confusion and uncertainty during volume management, which undermines the effectiveness and adherence of their management strategies. These findings suggest that healthcare providers should place greater emphasis on improving the health literacy of patients with lower education levels. The use of plain language and visual aids may support patient comprehension and foster more effective engagement in volume management practices.

#### 4.2.2 Course of disease

The results of this study indicate that, compared to patients with a disease course exceeding 5 years, those with a shorter duration of heart failure are more likely to be categorized as “low capacity-vulnerable type”. Patients with a longer disease course often accumulate and internalize relevant management knowledge and self-regulation strategies over extended periods of disease experience and medical interactions (34). In contrast, patients with a shorter disease course are typically at an early stage of disease cognition and psychological adaptation. Their understanding of volume management remains largely conceptual and has yet to be translated into stable behavioral habits (35). This highlights that the transformation from knowledge to behavior does not occur automatically. Patients need to gain experience through sustained disease management to develop lasting healthy behaviors. Therefore, it is recommended that healthcare providers offer repetitive and structured health education to newly diagnosed patients. Post-discharge, personalized guidance, online training, and continuous nursing support can be provided through the “Internet + platform” model to help patients translate the concept of volume management into sustainable daily practices.

#### 4.3.3 Social support

The findings suggest that patients with higher social support scores are more likely to belong to the “high capacity-robust group” or “moderate capacity-dependent group” compared to the C1 group. Strong social support enhances patients’ confidence and sense of control in managing their disease (36). The family is the most vital and widespread source of support, playing a crucial role in patients’ daily care, symptom monitoring, and health decision-making (37). However, CHF patients also face challenges due to a lack of external resources. Currently, community healthcare institutions in China face challenges such as inadequate human resource allocation and adequate facilities, which hinder the transformation of social support resources into effective caregiving capacity (38). Therefore, future nursing interventions should prioritize the integration of family resources and actively encourage family involvement in the patient's volume management. Simultaneously, it is crucial to strengthen the service capacity of community healthcare institutions. By enhancing the capabilities of these facilities, we can better address the diverse support needs of CHF patients throughout their disease management journey.

#### 4.3.4 Self-efficacy

The findings of this study suggest that individuals with lower self-efficacy scores are more likely to belong to the “low capacity-vulnerable type” compared to those in the “high capacity-robust type” and “moderate capacity-dependent type”. This may be attributed to the fact that patients with higher self-efficacy levels exhibit greater confidence in volume management. They are better equipped to proactively address challenges in disease management and maintain consistent capacity management behaviors (39). In contrast, patients with low self-efficacy tend to adopt negative attitudes toward volume management. They often fail to consistently monitor and manage their volume status consistently, engage in negative behaviors such as delayed medical visits, and refuse to participate in volume management, thereby increasing the risk of volume imbalance (40). Therefore, healthcare providers should implement various interventions aimed at enhancing self-efficacy in patients with heart failure. These interventions could include positive reinforcement, sharing success stories, or demonstrating the beneficial outcomes of fluid management. Such strategies will help strengthen patient confidence and willingness to participate, ultimately promoting sustained self-management behaviors.

## 5 Conclusion

This study utilized latent profile analysis to investigate the heterogeneity of volume management behaviors in patients with chronic heart failure. The findings revealed three distinct latent profiles of volume management behaviors: “low capacity-vulnerable type”, “high capacity-robust type”, and “moderate capacity-dependent type”. The influencing factors for these categories include self-efficacy, social support, educational level, and course of disease. Moving forward, healthcare professionals can develop personalized intervention strategies based on the characteristics of these categories and their associated influencing factors. Such targeted interventions can enhance patients’ volume management capabilities and ultimately contribute to improved prognosis in patients with chronic heart failure.

## 6 Limitations

First, this study is cross-sectional, meaning its findings can only reveal associations between variables, without inferring dynamic processes or establishing causal relationships over time. Second, the use of convenience sampling introduces the potential for selection bias. Additionally, the limited sample size and single-source participant pool resulted in inadequate geographical representation, thus limiting the generalizability of the results. Finally, the study did not fully account for potential confounding factors, such as psychological variables, which may have influenced the findings. Future research should enhance the applicability of results by increasing sample size, adopting a multicenter design, and employing more rigorous randomized sampling strategies. Longitudinal study designs are also recommended to track patients’ volume management behaviors and explore their developmental trajectories over time. Lastly, subsequent studies should integrate various potential confounding factors into analytical models to comprehensively assess the multifaceted influences on volume management behaviors in patients with chronic heart failure.

## Data Availability

The datasets presented in this article are not readily available because the dataset generated and analyzed in the current study contains personal health information of patients with chronic diseases, its use is subject to strict confidentiality agreements. For privacy and ethical considerations, these data cannot be publicly accessed. Requests to access the datasets should be directed to Xiaoyun Xiong, xxy6692@163.com.
